# Resection of Large Metachronous Liver Metastasis with Gastric Origin: Case Report and Review of the Literature

**DOI:** 10.7759/cureus.814

**Published:** 2016-10-03

**Authors:** Ionut Negoi, Alexandru Runcanu, Sorin Paun, Ruxandra Irina Negoi, Mircea Beuran

**Affiliations:** 1 General Surgery Department, Emergency Hospital of Bucharest, Carol Davila University of Medicine and Pharmacy Bucharest; 2 Anatomy and Embryology Department, Emergency Hospital of Bucharest, Carol Davila University of Medicine and Pharmacy Bucharest

**Keywords:** liver metastases, gastric cancer, metachronous resection, oligometastases

## Abstract

Introduction: Increasing evidence suggests that surgical resection may be offered to a subgroup of patients with liver metastasis of gastric adenocarcinoma. The aim of this case report is to illustrate the surgical resection of a single liver metachronous recurrence twelve months after a radical total gastrectomy for cancer.

Case report: A 63-year-old male patient with an Eastern Cooperative Oncology Group performance status of 1 was referred to our hospital for a single, large liver metastasis, twelve months after a radical total gastrectomy and DII lymphadenectomy for upper third gastric adenocarcinoma. As the adjuvant treatment, the patient received 12 cycles of FOLFOX chemotherapy. During the present admission, the abdominal computed tomography (CT) revealed a single liver metastasis located in the segments 5 and 6, of 105/85 mm in diameter. Surgical resection by an open approach of liver metastasis was decided. We performed a non-anatomical liver resection, without inflow control due to significant peritoneal adhesions in the liver hilum secondary to the previous lymphadenectomy. The patient was discharged after seven days, with an uneventful recovery. Six months after the second surgical procedure, the patient developed a local liver recurrence. The surgical resection of the liver recurrence was performed, with no postoperative morbidities, and the patient was discharged after eight days. Three months after the latest surgery, the patient is under adjuvant chemotherapy, with no imagistic signs of further recurrences.

Conclusions:  Hepatic resection for liver metastasis of gastric origin may offer satisfactory oncological outcomes in a very selected subgroup of patients.

## Introduction and background

Gastric cancer continues to be an aggressive malignancy, with 107,000 deaths in 2012, representing the fourth cause of mortality from cancer in Europe [1]. In western countries, most of the patients with gastric cancer are diagnosed in at advanced stage, and this may explain the five-year overall survival of 25%, compared to 70% reported in Japan [2]. Patients with metastatic disease present a dismal prognosis, with a reported median survival of four months in the case of best supportive care, and up to twelve months in the case of palliative chemotherapy [3]. Liver metastases from gastric cancer are present in 40% of patients dying from this disease [4]. However, these are rarely eligible for surgical resection, usually being multicentric or associated with extra-hepatic disease [5]. Data from retrospective studies with a relatively small number of subjects suggest that a subgroup of patients with gastric cancer and liver metastasis can be selected for surgery [6].

The objective of this case report is to illustrate the surgical resection of a liver metachronous recurrence twelve months after a radical resection for gastric cancer.

## Review

### Case report

**Figure 1:**
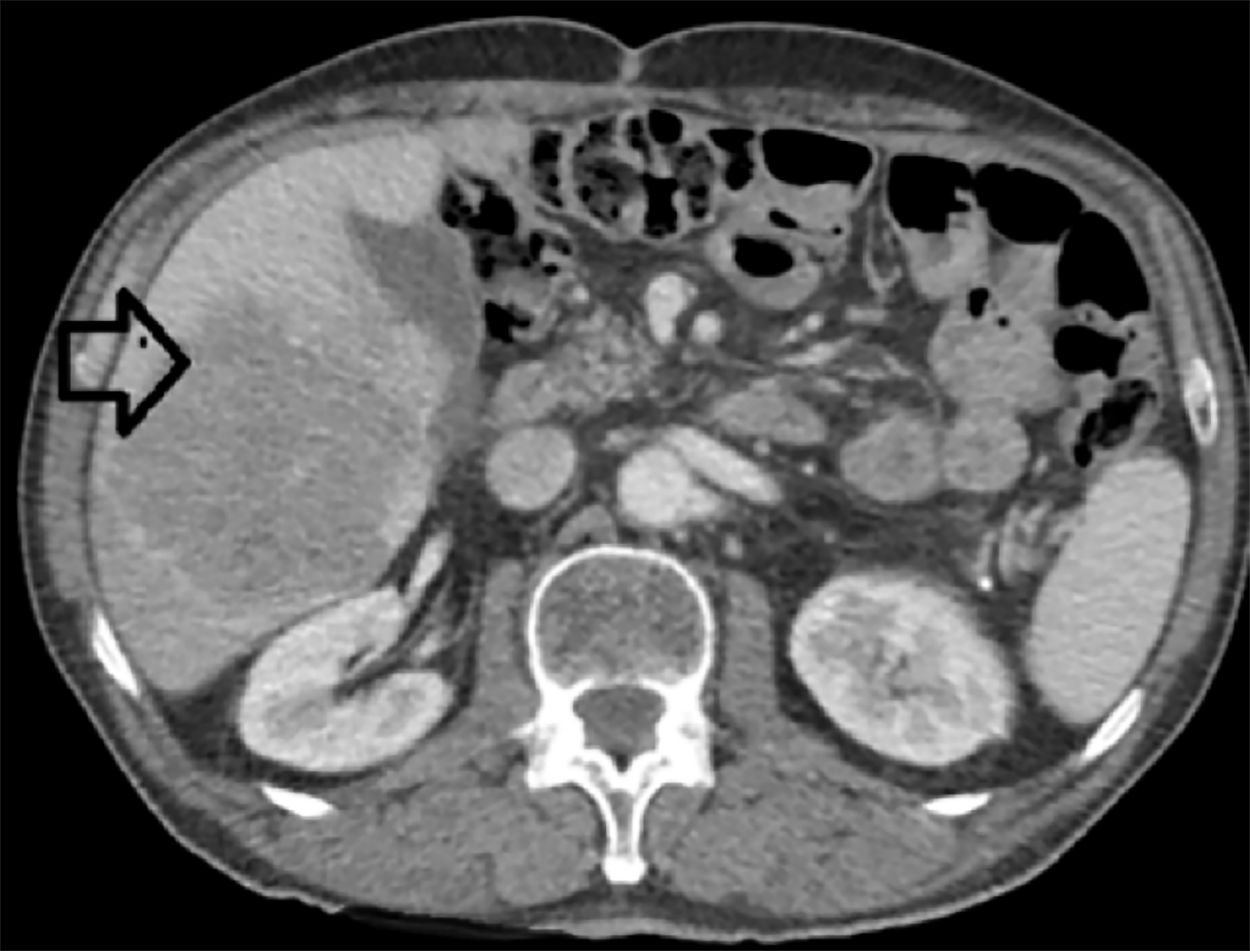
Computed Tomography Image Revealing a Single Liver Metastasis, Located in Segments 5 and 6 of the Liver

A 63-year-old male patient with an Eastern Cooperative Oncology Group index of 1 was referred to our hospital for a single, large liver metastasis, twelve months after a radical total gastrectomy and DII lymphadenectomy for upper third gastric adenocarcinoma. The initial pathology report identified a pT3N1M0LV1, moderate-differentiated, gastric adenocarcinoma. As an adjuvant treatment, the patient received 12 cycles of FOLFOX chemotherapy. During the present admission, the abdominal computed tomography (CT) revealed a unique liver metastasis located in the segments 5 and 6, of 105/85 mm in diameter (Figure 1).

**Figure 2:**
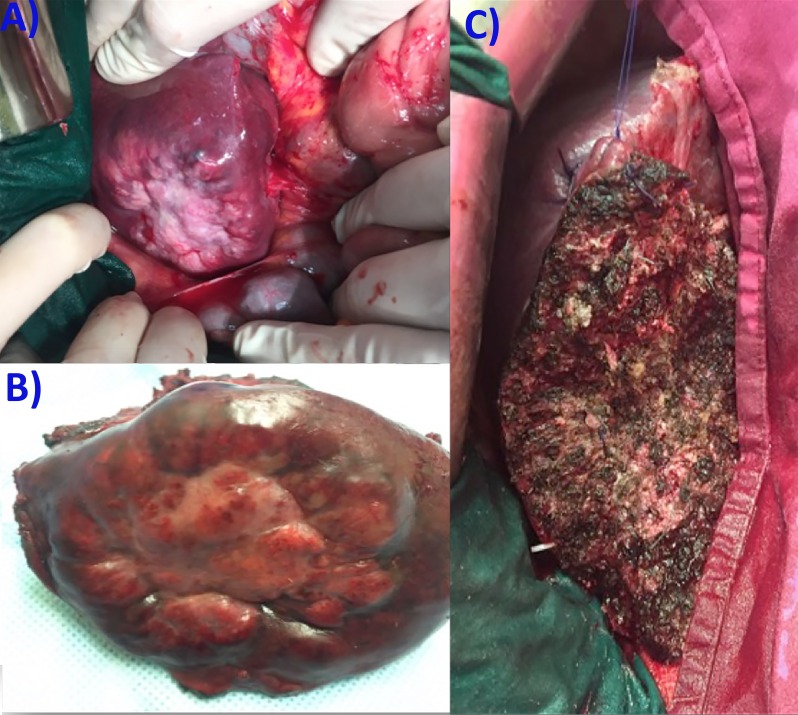
Intraoperative Aspects (A) the liver metastasis, (B) the resection specimen, and (C) the raw surface of the liver.

The 18-FDG PET-CT scan revealed no extrahepatic disease. Surgical resection by an open approach of liver metastasis was decided. The peritoneal washing revealed no malignant cytology, and intraoperative ultrasonography showed no additional liver metastatic disease. We performed a non-anatomical liver resection, without inflow control due to significant peritoneal adhesions in the liver hilum, secondary to previous lymphadenectomy (Figure 2).

The patient was discharged after seven days, with an uneventful recovery. The pathology report confirmed metastatic disease with a gastric origin. Six months after the second surgical procedure, the patient developed a local liver recurrence, of 84/73 mm diameter. The thoracic, abdominal, and pelvic CT revealed no extrahepatic metastases. The surgical resection of the liver recurrence was performed, with no postoperative morbidities, and the patient was discharged after eight days. Three months after the latest surgery, the patient is under adjuvant chemotherapy, with no imagistic signs of additional recurrences. 

### Discussions

We presented a case of a surgical resection for a unique liver metachronous recurrence twelve months after a radical resection for gastric cancer. 

Twenty-five to thirty percent of patients with radical gastrectomy for cancer will develop a metachronous liver metastasis, increasing the overall rate of hepatic involvement to 50% [7]. The liver is the most common site for metastases in patients with gastric cancer. However, the reported rate of liver resection for gastric cancer liver metastasis is less than 1%, due to the multiple, bilateral, and extrahepatic nature of the disease [8-9]. In high-volume tertiary centers, the hepatectomy for gastric cancer metastasis is performed in 7% of cases, compared to 12% for all types hepatic malignancies [10].

Oligometastases, usually defined as fewer than five metastases, should be differentiated from a more advanced state of the disease, with a significant metastatic burden [11-12]. Increasing evidence presents the oligometastatic state as an intermediate status, between localized stage and polymetastatic cancer [13]. The oligo-recurrence was defined as five or less metastatic or recurrent lesions, with a controlled primary tumor [14]. Our patient presented a single, large metastasis, diagnosed twelve months after the primary tumor resection. In this subgroup of patients, local therapies for metastatic disease may offer survival benefits, comparable with those for localized disease [13]. Lussier et al., identified patterns of microRNA expression in oligometastatic patients treated with high-dose radiotherapy [15]. They showed that microRNA-200c enhancement in an oligometastatic cell line resulted in polymetastatic progression [15]. Uppal et al., reported four microRNAs encoded in the 14q32 locus (microRNA-127-5p, microRNA-369-3p, microRNA-544a, and microRNA-655-3p) that are associated with oligometastatic phenotype [16]. These oligomiRs have overlapped biological effects that converge on cell proliferation, migration, adhesion, and epithelial to mesenchymal transition [16-17]. 

Reddy et al., evaluated the outcomes of partial hepatectomy in 82 patients with non-colorectal non-neuroendocrine (NCRNE) liver metastases [18]. Only one of these patients had a gastric cancer metastasis. The authors reported a longer median overall survival for neuroendocrine (NE) metastases, but similar for NCRNE and colorectal (CR) metastases (44 months for both). The multivariable analysis showed that delay of a liver resection for at least six months and chemoradiotherapy after resection might be associated with an improved overall survival (OS) [18]. Martel et al., presented the results of a multicenter study which included 52 patients (seven with gastroesophageal origin) with non-colorectal, non-neuroendocrine, and non-sarcoma metastasis [19]. The five-year overall survival for patients with a liver metastasis of gastro-esophageal origin was 50%, compared to 63% for a contemporary colorectal liver metastasis cohort [19]. Adam et al., developed a prognostic model for hepatic resection for NCRNE metastasis, starting from 1452 patients from 41 French centers [20]. R0 resection was obtained in 83% of cases, with a major complication rate of 21.5% and a 60-day mortality of 2.3%. 67% of patients have a tumor recurrence (24%-liver, 18%-extrahepatic, 25%-both). Factors associated with poor prognosis were: age > 60 years, non-breast origin, melanoma or squamous histology, disease free interval less than twelve months, extrahepatic metastases, R2 resection, and major hepatectomy [20]. Our patient had a local liver recurrence, diagnosed six months after the second surgery which was also amenable to surgical resection. Uggeri et al., published a systematic review of 30 articles, including 3849 patients, with surgical resection for non-colorectal, non-neuroendocrine and non-sarcoma metastasis [21]. The five-year OS of patients with liver metastasis of gastric and duodenal origin was 15%-30%. A worse five-year OS of less than 15% was observed in liver metastases of esophageal, cardiac, and pancreatic origin. Better outcomes were observed for liver metastasis with primary tumor located in the breast, kidney, uterus, ovary, ampulla of Vater, or adrenal glands, with a five-year OS greater than 30% [21]. A review of 420 NCRNE patients from four major liver centers showed a recurrent disease in 66% of patients [22]. The multivariable analysis found as independent predictors of poorer survival the lymphovascular invasion and metastases ≥ 5 cm. Although survival was not significantly different between various primary locations, it was significantly higher for cases managed between 2000-2009 compared with 1990-1999 (66 versus 32 months, P=0.003) [22]. The analysis of 106 patients from five hepatobiliary and pancreatic centers from Argentina revealed that curative resections and metachronous disease were predictors for better survival [23]. Earle et al., showed that a longer interval of time between primary surgery and diagnoses of liver relapse is predictive of survival [24].

Takemura et al., reviewed 73 patients with surgical resection of gastric cancer liver metastases [25]. They showed one-, three-, and five-year survival rates for macroscopically complete resections of 84.5%, 50%, and 37%, respectively [25]. Two hundred fifty-seven patients with gastric cancer liver metastases from five Japanese centers, showed a mean number of resected tumors of two, with a surgical mortality of 1.6% [26]. Seventy-five percent of patients had disease recurrence, especially in the liver (72.4%) after a median interval of seven months [26]. It should be noted that even long-term survival may be obtained for very selected patients. Out of 42 patients presented by Koga et al., eight survived more than five years [27]. However, only a small subgroup of liver metastases of gastric origin may be selected for surgical resection. From 319 patients with Stage IV gastric cancer, only 17 were resected [28]. A multicentric Italian survey presented 73 patients with metachronous hepatic metastases from gastric carcinoma [29]. The three-year survival rate was 2.2% in the supportive care group, 6.4% in the chemotherapy group, and 20.2% in the surgical group [29]. Markar et al., published the most recent meta-analysis regarding hepatectomy for metastases of gastric adenocarcinoma [30]. Thirty-nine studies published between 1990 – 2015 were included [30]. The pooled 30 morbidity and mortality were 24% (0%-47%) and 0% (0%-30%), respectively. The one-, three-, and five-year survival rates were 68%, 31%, and 27%, respectively. Surgical resection was associated with significant overall survival (hazard ratio = 0.5, P<0.001), especially for solitary lesions (odds ratio = 0.31, P=0.011) [30]. The were no differences between synchronous and metachronous metastasis resections (P=0.631) [31]. Another meta-analysis of 870 patients coming from 23 studies showed a five-year overall survival rate of 30% (95% confidence interval (CI) 24.7% to 35.8%) and 22.6% (95% CI 14% to 34.4%) for metachronous and synchronous resections, respectively [32]. The parameters associated with poorer survival were multiple metastases and large size of the metastases (see Table 1).

**Table 1:** Studies Including Patients with Surgical Resection for Gastric Cancer Liver Metastasis OS – overall survival, * – results of multivariable analysis, GC – gastric cancer

Study	Country	Time Interval	Number of Patients/Metachronuos Metastasis	5-year OS	Predictive Factors for Poorer OS
Okano et al., 2002 [33]	Japan	1986-1999	19/6	34%	More than one nodule, Synchronous disease
Koga et al., 2007 [27]	Japan	1985-2005	42/22	42%	*Serosal invasion of primary GC, *More than one nodule
Takemura et al., 2012 [25]	Japan	1993- 2011	73 (R0/R1 – 64)/32	37%	*Serosal invasion of primary GC, *Hepatic tumor > 5 cm
Garancini et al., 2012 [34]	Italy	1998-2007	21/9	19%	More than one nodule, Positive margin, Lack of peritumoral fibrous capsule
Aoyagi et al., 2013 [28]	Japan	1984- 2010	17/5	17.60%	
Qiu et al., 2013 [35]	China	1998- 2009	25/0	29.40%	*More than one nodule
Kinoshita et al., 2015 [26]	Japan	1990- 2010	256/150	31.10%	*Serosal invasion of primary GC, *³3 liver metastases, *Hepatic tumor > 5 cm

Although the chemotherapy represents the mainstay of treatment for liver metastases of gastrointestinal malignancies (and for very selected cases surgical resection may be taken into account), it seems that stereotactic body radiation therapy (SBRT) may offer local control with acceptable toxicity [36]. Mendez Romero et al., reported 34 metastases--none with gastric origin, and 11 hepatocellular carcinomas treated with SBRT. Local control rates were 94% and 82% at one and two years, respectively [36]. Rusthoven et al., evaluated SBRT in a multi-institutional study which included 47 patients with 63 liver metastases [37]. The one- and two-year local control rates were 95% and 92%, respectively, with a median survival of 20.5 months [37].

## Conclusions

Based on our case report and previously reported data, we conclude that hepatic resection for liver metastasis of gastric origin may offer better oncological outcomes in a very selected subgroup of patients.
